# Supplementary material for: Flexible Piezoresistive Polystyrene Composite Sensors Filled with Hollow 3D Graphitic Shells
Source: Polymers (Basel). 2023 Dec 11;15(24):4674. doi: 10.3390/polym15244674 (PMC10747410; doi:10.3390/polym15244674)
Supplement: Supplementary file 1 [file polymers-15-04674-s001.zip › polymers-2718465-supplementary.pdf]

polymers

MDPI

Supplementary Information

Flexible Piezoresistive Polystyrene Composite Sensors Filled with Hollow 3D Graphitic Shells

Nataliia Guzenko [1,2], Marcin Godzierz [1,3,*], Klaudia Kurtyka [1,3], Anna Hercog [1], Klaudia Nocoń-Szmajda [1], Anna Gawron [1,4], Urszula Szeluga [1,3], Barbara Trzebicka [1,3], Ruizhi Yang [5] and Mark H. Rümmeli [1,5,6,7,*]

[1] Centre of Polymer and Carbon Materials, Polish Academy of Sciences, M. Curie-Skłodowskiej 34, 41-819 Zabrze, Poland; guznataliia@gmail.com (N.G.); kkurtyka@cmpw-pan.pl (K.K.); ahercog@cmpw-pan.pl (A.H.); knocon@cmpw-pan.pl (K.N.-S.); agawron29@wp.pl (A.G.); uszeluga@cmpw-pan.pl (U.S.); btrzebicka@cmpw-pan.pl (B.T.)
[2] Chuiko Institute of Surface Chemistry, National Academy of Sciences of Ukraine, General Naumov Str. 17, 03164 Kyiv, Ukraine
[3] International Polish-Ukrainian Research Laboratory ADPOLCOM, 41-800 Zabrze, Poland
[4] Faculty of Biomedical Engineering, Silesian University of Technology, Roosevelta 40 Street, 41-800 Zabrze, Poland
[5] Key Laboratory of Advanced Carbon Materials and Wearable Energy Technologies of Jiangsu Province, Soochow Institute for Energy and Materials Innovations, College of Energy, Soochow University, Suzhou 215006, China; yangrz@suda.edu.cn
[6] Leibniz Institute for Solid State and Materials Research Dresden, P.O. Box 270116, D-01171 Dresden, Germany
[7] Institute of Environmental Technology, Centre for Energy and Environmental Technologies, VSB—Technical University of Ostrava, 17. Listopadu 15, 708 33 Ostrava, Czech Republic
* Correspondence: mgodzierz@cmpw-pan.pl (M.G.); mhr1967@yahoo.com (M.H.R.)

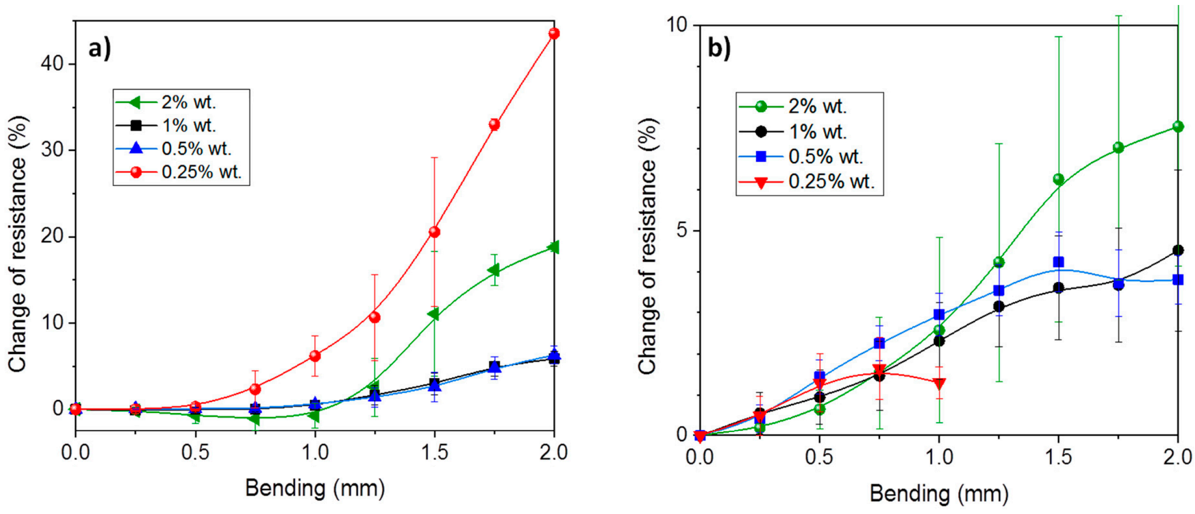

Figure S1. Change of resistance as a function of bending for GS/PS (a), CNT/PS (b). Results obtained by commercially available DPM Solid multimeter.

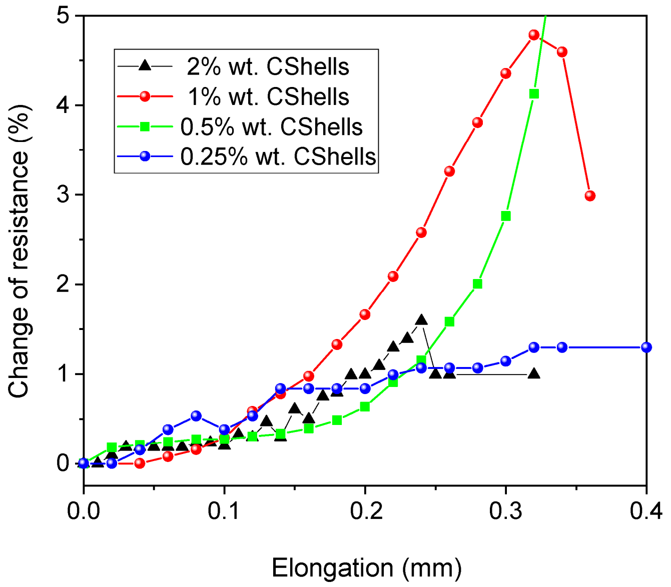

Figure S2. Change of resistance as a function of elongation for PS/3D-Graphene. Results obtained by commercially available DPM Solid multimeter.
